# Chitosan-Modified Gold Nanoparticle-Based Electrochemical Immunosensor for C-Reactive Protein Detection

**DOI:** 10.3390/bioengineering13060592

**Published:** 2026-05-22

**Authors:** Bilal Ahmad, Changyun Quan, Xiyue Zhang, Haiyan Xia, Zhenhong Yuan, Chenghua Zhu, Yang Zhang, Haixia Yang, Xueqin Huang, Chunyi Tong, Bin Liu, Binjie Xu

**Affiliations:** 1 College of Biology, Hunan University, Changsha 410000, China; bilalahmad_271@live.com (B.A.); sw_tcy@hnu.edu.cn (C.T.); 2 Cofoe Medical Technology Co., Ltd., No. 87, Section 1, Huanbao East Road, Changsha 410000, China; quanchangyun@163.com (C.Q.); zhangxiyue0920@163.com (X.Z.); xiahy2023@163.com (H.X.); yuanzh961221@163.com (Z.Y.); tw535390882@163.com (C.Z.); zyy388566@163.com (Y.Z.); yanghaixia@cofoe.com (H.Y.); huangxueqin@cofoe.com (X.H.); 3 Hunan Provincial Key Laboratory of Micro & Nano Materials Interface Science, College of Chemistry and Chemical Engineering, Central South University, Changsha 410000, China

**Keywords:** C-reactive protein, gold nanoparticles, electrochemical impedance spectroscopy, immunosensor, point-of-care-testing

## Abstract

C-reactive protein (CRP) is one of the most essential biomarkers for the early detection of inflammation and infection. In this study, we developed a sensitive and selective electrochemical immunosensor for CRP detection, leveraging the unique properties of gold nanoparticles (AuNPs). A nanostructured layer of AuNPs was deposited onto a screen-printed carbon electrode (SPCE), followed by the formation of a self-assembled monolayer (SAM) of L-cysteine and EDC/sulfo-NHS chemistry. The antibody was covalently immobilized onto the modified electrode through optimized dual-crosslinking chemistry. Detection conditions were systematically optimized, with pH 8.0 in Tris buffer providing the best electrochemical response. Electrochemical characterization was performed using cyclic voltammetry (CV), electrochemical impedance spectroscopy (EIS), and differential pulse voltammetry (DPV) in a 5 mM K_3_[Fe(CN)_6_]/K_4_[Fe(CN)_6_] redox probe solution containing 0.1 M KCl. CRP detection was achieved by monitoring the increase in charge transfer resistance (Rct) upon specific binding of the target CRP antigen to the immobilized antibody. Spiked recovery experiments showed spiked recovery rates ranging from 98.01% to 107.14%, with a standard deviation below 4%. Regeneration studies demonstrated high efficiency, confirming the suitability of the sensor interface for repeated and reliable measurements. Under optimized conditions, the immunosensor exhibited excellent analytical performance, including a low limit of detection (LOD) of 0.16 µg/mL, a wide linear detection range of 5–100 µg/mL, high selectivity against 13 potential interferents (including inflammatory cytokines), and good reproducibility with a relative standard deviation (RSD) of 3.69%. The sensor also showed strong stability, retaining more than 95% of its signal after 15 days, and high regeneration efficiency of 97% over seven cycles. These results highlight the strong potential of the proposed immunosensor for point-of-care (POC) applications due to its simple fabrication, cost-effectiveness, user accessibility, and robust analytical performance.

## 1. Introduction

CRP is an essential protein biomarker for the early detection of inflammation after acute infection. During bacterial infections, autoimmune disorders, and cardiovascular illnesses, quick and substantial elevation of CRP concentrations in response to diverse inflammatory stimuli renders CRP a crucial diagnostic and prognostic biomarker [1]. CRP is produced by the liver in response to inflammatory cytokines, especially interleukin-6 (IL-6). The amount of CRP in the blood can increase dramatically (up to 1000-fold) within hours during the inflammation. This dramatic increase makes CRP a sensitive and early sign of inflammation. The reference level of CRP in the blood of healthy individuals typically falls below 3 µg/mL. Levels between 0 and 1 µg/mL are considered low-risk, 1 to 3 µg/mL indicates intermediate risk, and levels between 3 and 10 µg/mL are associated with high risk for potential cardiovascular events. Levels of CRP between 10 and 50 μg/mL are considered viral infections, while those between 50 and 200 μg/mL are generally deemed to be bacterial infections. CRP values above 200 μg/mL are relatively infrequent, indicating serious health issues in affected individuals [2,3]. Traditional laboratory-based CRP assays are precise, but mostly require dedicated equipment and professional staff, and a prolonged waiting time for result acquisition. This limitation highlights the necessity for rapid, precise, and economical POC testing methods for CRP detection, particularly in limited-resource environments and during emergency [4].

Electrochemical techniques are widely used due to their high sensitivity, fast analysis time, and requirement of cost-effective equipment. This technique only requires a small volume of sample [5]. Electrochemical biosensors are becoming useful tools for clinical diagnostics because they are exceedingly sensitive, quick, portable, and affordable. These biosensors can check CRP levels in real time, which makes it possible to find sepsis early and monitor it all the time. A redox mediator such as [Fe(CN)_6_]^3−/4−^ is used to test the electrochemical current response changes in label-free electrochemical immunosensors for CRP detection. The development of an immunocomplex between the target CRP and anti-CRP antibodies on the electrode surface is what triggered this alteration [6].

Some studies demonstrated the limitations in the sensitivity of label-free electrochemical immunosensors for CRP detection [7]. Various nanomaterials, including carbon nanotubes, AuNPs, quantum dots, graphene, and metal oxide nanoparticles, can be used to make CRP immunosensors that work better and are more sensitive [8,9]. Due to facile synthesis, colloidal stability, diverse surface functionalization, and plasmonic characteristics, AuNPs have been significantly used in immunoassays [10,11]. AuNPs have a unique physical and chemical property that makes them useful for electrochemical biosensing. Their high surface-to-volume ratio provides a large active area for binding biomolecules like antibodies, enzymes, or DNA probes. This increases the number of recognition sites and makes the sensor work better overall [12,13]. Similarly, AuNPs’ higher electrical conductivity speeds up the transport of electrons between the biological recognition molecules and the electrode surface area, which is necessary for high sensitivity and quick signal transduction [13]. A significant benefit of AuNPs is that they are incredibly biocompatible. They are stable with biomolecules and beneficial for a long time in biosensing circumstances since they interact positively with biological systems without causing harm. This compatibility ensures that the antibodies or biomolecules that are stuck in place preserve their inherent activity and ability to attach [14]. Integrating AuNPs into electrochemical immunosensors significantly increases the analytical performance of the immunosensor by enhancing sensitivity, lowering detection limits, accelerating reaction times, and improving consistency. AuNP-modified electrodes are now one of the most common and important components of modern biosensor design due to their substantial advantages [15].

Herein, we developed a label-free electrochemical immunosensor for CRP detection using screen-printed carbon electrode arrays functionalized with AuNPs and SAM. The antibody was covalently bonded to the surface of the SAM layer through amide crosslinking. The CRP antigen was measured by changes in charge transfer efficiencies between the electrode and redox probe [Fe(CN)_6_]^3−/4−^ using electrochemical techniques such as CV, EIS, and DPV. The effects of different factors that affect the sensitivity of detection were also studied. Moreover, analytical performance metrics, including sensitivity and specificity, of the proposed immunosensors were also evaluated. This immunosensor was ultimately developed to measure CRP in verified human serum samples to assess its efficacy in quantifying CRP levels. The results demonstrated that this immunosensor offers great sensitivity, stability, reproducibility, reusability, and selectivity for CRP detection, free from interference. The constructed immunosensor has a clinically significant detection threshold, an extensive dynamic range, elevated specificity, and commendable storage stability at room temperature. These attributes facilitate their effective implementation in point-of-care applications, particularly in resource-limited or decentralized clinical settings.

## 2. Materials and Methods

### 2.1. Chemicals and Instruments

Bovine serum albumin (BSA), hemoglobin, chloroauric acid trihydrate (HAuCl_4_.3H_2_O), and bilirubin were purchased from Sigma Aldrich, Taufkirchen, Germany. Potassium ferricyanide K_3_[Fe(CN)_6_] was acquired from Macklin, Shanghai, China. Sodium chloride, disodium hydrogen phosphate, citric acid (CA), and potassium dihydrogen phosphate were received from Sinopharm Chemical Reagent Co., Ltd., Shanghai, China. Potassium ferrocyanide K_4_[Fe(CN)_6_], potassium chloride (KCl), Tris, glutaraldehyde 50%, 3,3′,5,5′-Tetramethylbenzidine (TMB), piperazine-N,N′-di (2-acesulfonic acid) PIPES, N-2-hydroxyethylpiperazine-N′-2-ethenesulfonic acid (HEPES), Morpholine ethysulfonic acid (MES), N-hydroxy succinimide (NHS), glucose, urea, glycine, ascorbic acid (AA), cysteine, and creatinine were obtained from Shanghai Aladdin Biochemical Technology Co., Ltd., Shanghai, China. 1-Ethyl-3-(3-dimethylaminopropyl) carbodiimide (EDC) was purchased from Thermo Scientific, Waltham, MA, USA. Recombinant human CRP, recombinant anti-C-reactive protein antibody, human serum albumin (HSA), interleukin-6 (IL-6), immunoglobin G (IgG), tumor necrosis factor-alpha (TNF-α), and horseradish peroxidase (HRP) were purchased from Sino Biological, Beijing, China. Hydrogen peroxide (H_2_O_2_) was acquired from Hunan Huihong Reagent Co., Ltd., Changsha, China.

The three-electrode electrochemical workstation CHI650E (CH Instruments, Austin, TX, USA) was used for CV, EIS, and DPV measurements. Surface plasmon resonance peaks were captured by UV-vis spectrophotometry, UV-250, Shimadzu, Columbia, MD, USA. Transmission electron microscopy (TEM) images were captured by Talos-F200S-G2, Thermo Fisher, Waltham, MA, USA. The size and zeta potential values of AuNPs were measured by Zeta Potential Meter, BeNano 90 Zeta, Dandong, China. Bruker Quantax XFlash SDD 6|30 Esprit 2.0, Karlsruhe, Germany was used for EDX analysis. Field emission scanning electron microscope (FESEM) Zeiss Sigma 300, Baden-Fürburg, Obercohen, Germany, was used for SEM analysis. Thermo Fisher Scientific K-Alpha, Waltham, MA, USA, was used for XPS analysis. Bruker TENSOR27, Karlsruhe, Germany, was used for Fourier-transform infrared spectroscopy (FTIR) spectrum.

### 2.2. Synthesis of AuNPs

AuNPs were synthesized using a modified Turkevich method, which involves the reduction of chloroauric acid (HAuCl_4_) using 1.5% trisodium citrate as both a reducing and stabilizing agent in 10 mL of water. In this procedure, a boiling solution (100 °C) of HAuCl_4_ was rapidly mixed with a specific concentration of sodium citrate under vigorous stirring, initiating a series of color changes from pale yellow to deep red, indicating nanoparticle formation. At elevated temperature (100 °C), citrate ions reduce Au^3+^ to Au^0^ and subsequently act as capping agents, stabilizing the synthesized nanoparticles. This results in the formation of negatively charged AuNPs, which prevents aggregation through electrostatic repulsion [16].

### 2.3. Characterization of AuNPs

AuNPs were characterized using various techniques to evaluate their physicochemical properties for specific applications. UV–vis spectroscopy was employed to monitor the surface plasmon resonance (SPR) peak, which typically appears around 522 nm for spherical AuNPs. TEM was used to determine particle size (typically 10–100 nm), morphology, and crystallinity with high-resolution imaging. DLS was utilized to measure the hydrodynamic diameter and polydispersity index (PDI). Zeta potential analysis was conducted to assess colloidal stability, with citrate-capped AuNPs typically exhibiting values in the range of −30 to −40 mV. XPS was used to determine the elemental composition and oxidation states of the nanoparticle surface, which is particularly important for functionalized AuNPs. FTIR was employed to identify surface functional groups, such as citrate or thiol ligands, enabling subsequent bioconjugation for biosensing applications.

### 2.4. Design and Fabrication of Immunosensor

**Figure 1:**
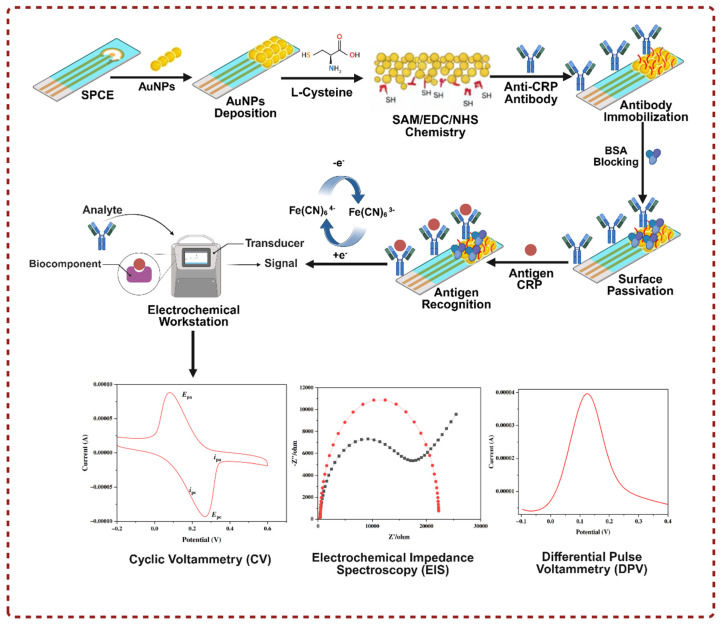
Schematic illustration of the stepwise development of the CRP immunosensing platform, showing electrode surface preparation, nanomaterial modification, antibody immobilization, antigen recognition, and final electrochemical detection of CRP.

The screen-printed carbon electrode was cleaned by sonication for 5 min each in ethanol and deionized water, followed by drying at room temperature. A 10 µL aliquot of chitosan (1 mg/mL in 1% acetic acid) was then drop-cast onto the electrode surface and allowed to dry at room temperature, forming a biocompatible, amine-rich polymer underlayer. The modified electrode was subsequently treated with 10 µL of glutaraldehyde (2% *v*/*v*) and incubated for 30 min at room temperature, resulting in the formation of covalent imine linkages between the aldehyde groups of glutaraldehyde and the free –NH_2_ groups of chitosan, thereby creating a mechanically stable three-dimensional network. After rinsing with phosphate-buffered saline (PBS) buffer, 10 µL of AuNPs was deposited onto the electrode surface and incubated to provide a conductive scaffold for subsequent self-assembled monolayer (SAM) formation. A 10 µL aliquot of L-cysteine (100 mM) was then drop-cast onto the electrode and incubated for 2 h at room temperature, allowing chemisorption of thiol groups onto the gold surface and exposing terminal carboxyl (–COOH) groups. These functional groups were activated by adding 10 µL of a mixture containing EDC (50 mM) and sulfo-NHS (80 mM) onto the modified electrode and incubating it for 30 min at room temperature. Specifically, EDC activates the –COOH groups to form a reactive O-acylisourea intermediate, which is subsequently stabilized by sulfo-NHS to generate a more stable sulfo-NHS ester under aqueous conditions. A 10 µL aliquot of anti-CRP antibodies (50 µg/mL) was then immobilized onto the activated surface through covalent amide bond formation with lysine –NH_2_ residues. Non-specific binding sites were subsequently blocked by incubating the electrode with 10 µL of BSA (0.5% *w*/*v*) for 60 min at room temperature. After rinsing with PBS, the BSA-blocked immunosensor was incubated with CRP at concentrations of 0, 5, 10, 20, 30, 50, and 100 µg/mL for 60 min at room temperature, followed by gentle rinsing with PBS. Electrochemical characterizations were performed using EIS, CV, and DPV in a solution containing 5 mM [Fe(CN)_6_]^3−^/^4−^ and 0.1 M KCl. The progressive increase in charge transfer resistance confirmed the successful layer-by-layer assembly of the immunosensor. A schematic illustration of the electrode fabrication and sensing mechanism is presented in Figure 1.

A sandwich-style CRP quantification assay was also performed, using a secondary antibody labeled with horseradish peroxidase and 0.003% hydrogen peroxide to increase the signal through a redox enzyme reaction. The immunosensor performance was evaluated by DPV, in which the primary antibody was immobilized onto modified electrodes to form an antigen–antibody combination with CRP. The secondary antibody was conjugated with HRP. In the presence of H_2_O_2_, HRP enzyme catalyzes the reduction of H_2_O_2_ to water in the presence of TMB (0.5 mg/mL), producing an electrochemical signal that is directly proportional to CRP concentrations [17]. The redox enzyme equation for horseradish HRP is as follows:

(1) HRP _(reduction)_ + H_2_O_2_ → HRP _(oxidation)_ + H_2_O

### 2.5. Electrochemical Measurements of Target CRP

Electrochemical techniques, including CV, EIS, and DPV, were employed for both qualitative and quantitative detection of CRP. CV measurements were performed over a potential range of −0.3 V to 0.9 V at a scan rate of 0.05 V/s, with a sensitivity of 1 × 10^−4^ A/V. EIS data were collected over a frequency range of 0.1 Hz to 100 kHz and, in some measurements, from 1 Hz to 100 kHz, with an applied AC amplitude of 0.005 V. DPV measurements were carried out using a pulse amplitude of 0.05 V within a potential window of −0.1 V to 0.4 V, with a sensitivity of 1 × 10^−4^ A/V. The CV and DPV responses of both bare and modified electrodes were used for qualitative analysis of CRP. EIS results were presented as Nyquist plots and analyzed using the Randles equivalent circuit model, with data fitting performed using ZView 4 and OriginPro software, 2024.

### 2.6. Specificity, Stability, and Reproducibility

Specificity studies of the proposed immunosensor were conducted by detecting CRP at a concentration of 10 µg/mL in the presence of potential interfering compounds, including ascorbic acid, citric acid, glucose, cysteine, glycine, urea, creatinine, bilirubin, hemoglobin, human serum albumin (HSA), interleukin-6 (IL-6), immunoglobulin G (IgG), and tumor necrosis factor-α (TNF-α), each tested at concentrations 10 times higher than that of CRP. The stability of the immunosensor was evaluated by storing it at 4 °C for 15 days, with periodic EIS measurements performed to monitor changes in performance over time. Reproducibility was assessed by using ten independently prepared electrodes by measuring the variation in charge transfer resistance at a low-frequency cutoff of 0.1 Hz upon CRP binding.

### 2.7. Regeneration Method of CRP Immunosensor

The regeneration study was initiated with an initial CRP binding step (Cycle 0), in which the CRP immunosensor was incubated with a 50 µg/mL CRP solution for 30 min at room temperature to facilitate antigen–antibody complex formation. Following incubation, the sensor was thoroughly rinsed with PBS to remove unbound CRP. DPV measurements were then performed in a redox solution of 5 mM [Fe(CN)_6_]^3−^/^4−^ to determine the initial peak current corresponding to the bound CRP. Subsequently, the regeneration step was carried out by immersing the immunosensor in Glycine–HCl regeneration buffer at concentrations of 0.2 M, 0.4 M, and 0.6 M for 60 s to induce dissociation of the bound CRP from the antibody binding sites. After regeneration, the sensor was thoroughly rinsed with PBS to remove any residual buffer and dissociated CRP. To evaluate regeneration efficiency and reusability, the regenerated immunosensor was reanalyzed in the redox solution using DPV. This process, including regeneration, re-incubation with CRP, and DPV measurement, was repeated for a total of seven cycles to assess the long-term performance and stability of the immunosensor under each regeneration condition.

## 3. Results and Discussion

### 3.1. Characterization of AuNPs and Modified Electrodes

TEM images of AuNPs clearly showed spherical and slightly irregular morphologies, which were characteristic of AuNPs synthesized through the proposed methods. The particles were between 20 and 100 nm in size, indicating good control of size. The nanoparticles were homogeneous because they had smooth surfaces and did not stick together too much [18]. Figure S1a showed that nanoparticles were clearly separated at higher magnifications. Overall, TEM pictures showed that AuNPs were well-dispersed and not clumped together, which showed that they were successfully synthesized and stable enough to be used in immunosensors.

The surface morphology of AuNPs was examined using FESEM at different magnifications corresponding to scale bars of 500, 200, and 100 nm (Figure S1b). At a 500 nm scale, AuNPs exhibited a porous structure with noticeable gaps between particle clusters. As the magnification increased to 200 nm, the particles appeared smaller and more closely packed, although some voids were still present, indicating a transition toward a more compact structure with better-defined surface features. At the highest magnification (100 nm), the nanoparticles appeared predominantly spherical and were densely packed with minimal interparticle voids, resulting in a smoother and more compact surface morphology. Overall, the observed morphological evolution demonstrates a transition from a loosely arranged porous structure to a densely packed configuration with decreasing particle size and increasing magnification [19].

**Figure 2:**
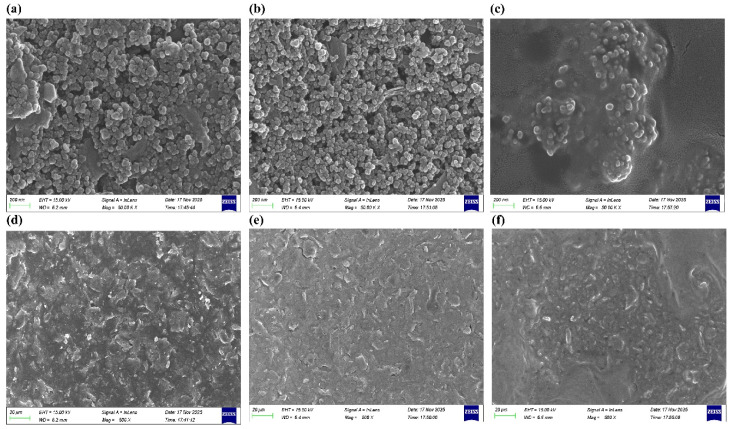
FESEM images related to the morphological changes of modified electrodes with different magnifications. (**a**) Bare electrode at 200 nm. (**b**) Antibody CRP at 200 nm. (**c**) Target analyte CRP at 200 nm. (**d**) Bare electrode at 20 µm. (**e**) Antibody CRP at 20 µm. (**f**) Target analyte at 20 µm.

Figure 2a and Figure 2d show the morphology of the bare screen-printed carbon electrode at different scale bars of 200 nm and 20 µm respectively. The surface has a rough, even, and mixed structure, with clear pores on the micro scale. This roughness makes the electrochemical sensing performance better due to the interaction of more surface area. EDX analysis (Figure S2e) corroborated the results. Au element peaks showed a weight percentage of 92%, which is much greater than other elements on the Au electrode. This indicates that gold (Au) is the main element in the bare electrode [7]. Figure 2b and Figure 2e show FESEM images of the anti-CRP antibody, with a scale bar of 200 nm and 20 µm, respectively. The images showed that the surface morphology gets smoother, which makes it look more uniform and consistent than the bare electrode [20]. Similarly, Figure 2c and Figure 2f show FESEM images after the binding of the target CRP at 200 nm and 20 µm, respectively. These morphological observations are presented as an auxiliary characterization of AuNP synthesis and surface deposition [19].

Figure S2a shows the XPS spectrum of AuNPs, which has several peaks that are typical of different elements. The significant peak at about 85 eV is related to the Au 4f orbital, which shows that gold is the main element of the nanoparticles [21]. A peak at 1070 eV shows Na 1s, which is related to the sodium element. The O 1s peak at about 530 eV shows the presence of oxygen, probably because there was a thin layer of oxide on the surface of the nanoparticle. The peak near 160 eV is related to S 2p, which shows that sulfur is present and suggests that the surface has been functionalized. The C 1s peak at 284 eV is unique to carbon and indicates calibration and small amounts of organic contaminants. The overall XPS results confirmed that gold is the primary component in AuNPs.

The FTIR spectrum shows significant images of the surface chemistry of the synthesized AuNPs (Figure S2b). The peak at 3444 cm^−1^ corresponds to O-H stretching vibrations, showing the presence of hydroxyl groups. This broad peak is most likely representing water molecules adsorbed on nanoparticle surfaces or hydroxyl groups from surface modification or manufacturing. The band at 2080 cm^−1^ is assigned to the linear Au^0^–CO carbonyl stretching vibration at low-coordination metallic gold surface sites, arising from residual CO generated during the oxidative decarboxylation of citrate in the Turkevich synthesis. The peak at 1639 cm^−1^ is an asymmetric carboxylate stretch of surface-coordinated citrate due to the C=C stretching vibration from aromatic rings, which suggests the presence of organic ligands such as thiols or amines on the nanoparticle surface. The peak at the 689 cm^−1^ is ascribed to C-H bending vibrations due to organic ligands attached to the surface of gold nanoparticles and carboxylate deformation of citrate. Overall, the FTIR spectrum confirmed the successful synthesis of citrate-stabilized AuNPs [22].

The zeta potential of AuNPs was measured at −31.16 mV, which indicated good colloidal stability (Figure S2c). The negative surface charge provided successful electrostatic stabilization, as identified by the citrate agents usually utilized in AuNP production [23]. DLS analysis revealed a monomodal size distribution with a peak intensity of 62.71 nm, indicating a homogeneous nanoparticle population (Figure S2d). The tight distribution curve also explained outstanding monodispersity, which is critical for applications that need uniform nanoparticle behavior.

The EDS spectrum validated AuNPs elemental compositions. Peaks at 2.1 keV and approximately 10 keV were attributed to gold (Au), indicating its existence in nanoparticles (Figure S2e). The spectrum also possessed many additional elements, such as sodium (Na), oxygen (O), chlorine (Cl), carbon (C), phosphorus (P), and sulfur (S), which have equivalent peaks at 1.0 keV, 0.5 keV, 2.6 keV, 0.3 keV, 2.0 keV, and 2.3 keV, respectively. The relative intensities of these peaks showed that gold is the predominant element, followed by significant quantities of sodium, chlorine, and other elements [24].

The successful functionalization of AuNPs with CRP was confirmed using UV-vis spectroscopy (Figure S2f). The visible characteristic peak, which was obtained after the surface plasmon resonance (SPR) band, was very sensitive to changes in the nanoparticles’ size, shape, and the dielectric environment around them. AuNPs have a maximum absorption peak at about 522 nm, which is normal for spherical gold nanoparticles. When CRP is modified with AuNPs to make the AuNPs@CRP bioconjugate, SPR band shifts noticeably increased. Also, the peak absorbance increased from 0.44 a.u. to 0.53 a.u. The monitored redshift in the SPR band and the increase in absorbance provided strong spectroscopic confirmation for the successful and stable conjugation of CRP and AuNPs [23].

### 3.2. Electrochemical Characterization of Modified Electrodes

The electrochemical characteristics of modified electrodes were analyzed using the EIS, DPV, and CV methods. EIS was employed to evaluate the charge transfer resistance between the redox probe [Fe(CN)_6_]^3−^/^4−^ and the modified electrode surface. The EIS results are presented as Nyquist plots, in which each data point corresponds to the impedance at a specific frequency, spanning from high to low frequency [25]. The semicircle diameter observed in the high-frequency region of Nyquist plots indicates the charge transfer resistance, which directed the electron-transfer kinetics of the redox probe, 5 mM [Fe(CN)_6_]^3−^/^4−^, at the electrode interface. The electrode modification steps were conducted in a solution containing 5 mM [Fe(CN)_6_]^3−^/^4−^ and 0.1 M KCl as redox probe. The EIS results are presented as Nyquist plots, where the negative imaginary impedance (−Z″) is plotted against the real impedance (Z′). The diameter of the semicircle in the high-to-medium frequency region corresponds to the charge transfer resistance, which serves as a key parameter for monitoring binding events at the electrode surface [26].

**Figure 3:**
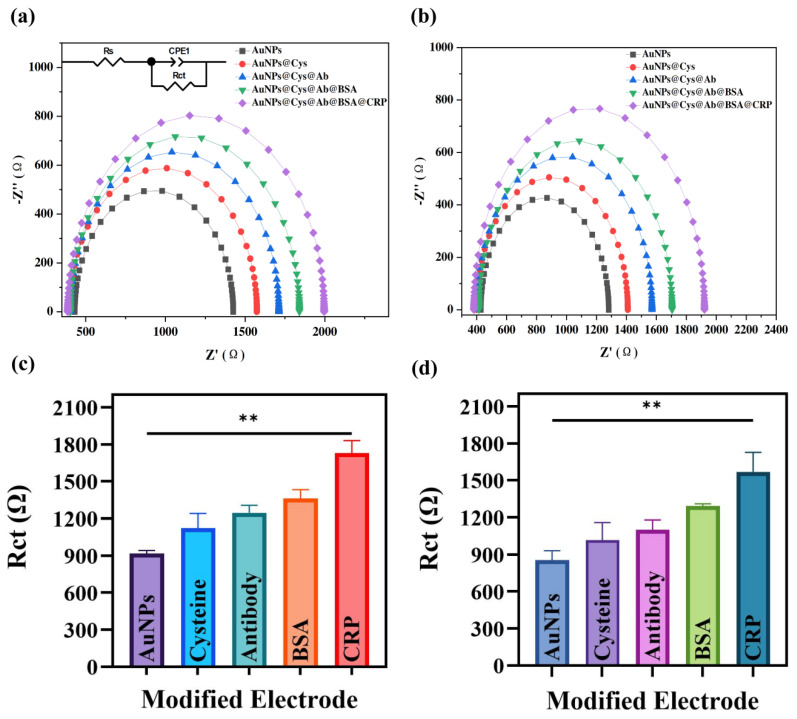
Electrochemical characterization of modified electrodes in electrolyte solution of 5 mM Fe [CN_6_]^3−/4−^ in 0.1 M KCl. (**a**) EIS Nyquist plot of AuNPs, AuNPs@Cys, AuNPs@Cys@Ab, AuNPs@Cys@Ab@BSA and AuNPs@Cys@Ab@BSA@CRP at low-frequency cutoffs of 0.1 and (**b**) 1 Hz. Inset is Randles’ equivalent circuit. (**c**) Quantitative representation of modified electrode with low-frequency cutoffs of 0.1 and (**d**) 1 Hz. Data are presented as mean ± standard deviation from three sets of data (*n* = 3) and ** denotes *p* < 0.01.

As shown in Figure 3a,c, the Nyquist plots demonstrate a consistent and significant increase in charge transfer resistance with each successive modification step, evaluated at a low-frequency cutoff of 0.1 Hz. The Rct values gradually increased from bare AuNPs (494 Ω) to the self-assembled monolayer (SAM) layer (587 Ω), followed by antibody immobilization (653 Ω), BSA blocking (716 Ω), and finally CRP binding (803 Ω) [27] The semicircles are visibly depressed and their centers lie slightly below the real axis, a hallmark of constant-phase element (CPE) behavior arising from the surface heterogeneity of the multilayer chitosan/GA/AuNPs/L-cysteine/Ab/BSA layer [28,29]. Accordingly, the data were fitted to the equivalent circuit Rs + (CPE∥Rct), in which the dense protein multilayer converts the electrode from a diffusion-accessible surface to one where electron-transfer kinetics are rate-limiting, suppressing the low-frequency Warburg tail that would otherwise appear on a bare or lightly modified electrode. All equivalent circuit fits yielded weighted chi-squared values (χ^2^) below the threshold that confirmed model adequacy and indicated that the selected equivalent circuit provides a reliable fit to the experimental EIS data. Solution resistance (Rs) was stable across all electrode modification steps [30,31]. The complete fitted parameters for all modified electrodes are provided in Table S1.

The Nyquist plots, recorded at a low-frequency cutoff of 1 Hz, exhibit an increase in charge transfer resistance, providing clear insight into the interfacial electron-transfer kinetics and confirming the successful fabrication of the sensor (Figure 3b,d). This increase indicates the successful covalent immobilization of anti-CRP antibodies onto the SAM-modified electrode surface. The results obtained at low-frequency cutoffs of 0.1 Hz and 1 Hz further confirmed the effective development of the proposed immunosensor. As shown in Figure 3c,d, the Rct values for different electrode modification steps were compared, with the CRP-modified electrode exhibiting the highest Rct. This suggests a significant increase in interfacial impedance and a substantial barrier to electron transfer due to antigen–antibody complex formation. Overall, these results demonstrate that an increase in Rct corresponds to higher impedance and reduced electron-transfer efficiency, with CRP binding producing the most pronounced effect among the tested modifications.

**Figure 4:**
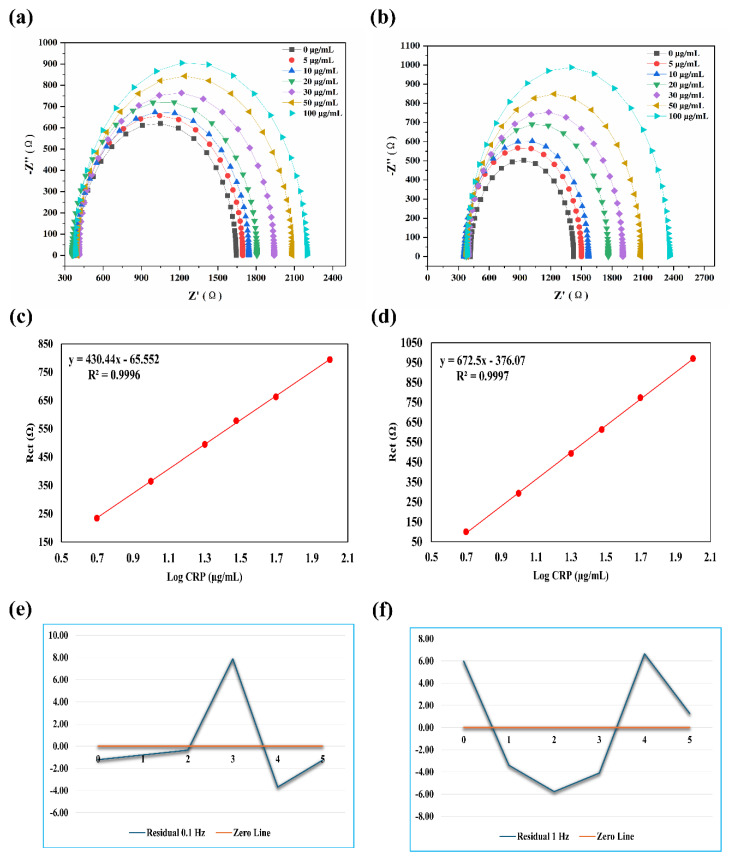
EIS Nyquist plot CRP at concentrations of 0, 5, 10, 20, 30, 50, and 100 µg/mL at low-frequency cutoffs (**a**) 0.1 and (**b**) 1 Hz. (**c**) Calibration linear fitting curves of different Log concentrations of CRP at 0.1 and (**d**) 1 Hz. Residual plot of semi-logarithmic calibration curves for CRP immunosensor at two EIS measurement frequencies (**e**) 0.1 and (**f**) 1 Hz. Data are presented as mean ± standard deviation from three sets of data (*n* = 3).

EIS was employed to evaluate the performance of the developed biosensor for CRP detection at different concentrations (0, 5, 10, 20, 30, 50, and 100 µg/mL). Figure 4a,b show Nyquist plots for the immunosensor incubated with increasing concentrations of CRP, ranging from 5 to 100 µg/mL. Figure 4a corresponds to measurements recorded with a low-frequency cutoff of 0.1 Hz, while Figure 4b represents measurements obtained at a low-frequency cutoff of 1 Hz; both extend to a high-frequency limit of 100 kHz. In both cases, a clear and systematic increase in the semicircle diameter is observed with increasing CRP concentration [32]. The binding of the target analyte to the immobilized antibody on the electrode surface created a non-conductive layer, which blocks the diffusion and electron-transfer kinetics of [Fe(CN)_6_]^3−^/^4−^ redox probe to the electrode surface. A comparison of Figure 4a,b shows that the overall shape and the trend of the Nyquist plots are highly consistent, representing the robustness of the immunosensor despite a minor variation in the low-frequency measurement range. The inclusion of lower-frequency cutoffs (0.1 and 1 Hz), which presents a more complete representation of diffusion-limited processes, accounts for the small difference in absolute charge transfer resistance values at the highest CRP concentration.

As shown in Figure 4c,d, a semi-logarithmic calibration was constructed by plotting the measured charge transfer resistance against the base-10 logarithm of CRP concentration (log_10_ CRP). The semi-logarithmic calibration format was selected based on the logarithmic dose response behavior commonly observed in label-free impedimetric immunosensors. Semi-logarithmic EIS calibration is widely used for impedimetric immunosensors for CRP and other protein biomarkers. It also compresses the clinically relevant CRP concentration range into a clear, readable plot, allowing better assessment of linearity and residual distribution [32,33,34]. A linear relationship between Rct and log_10_ CRP was established over the validated concentration range of 5–100 µg/mL, yielding R^2^ = 0.9996 at 0.1 Hz and 0.9997 at 1 Hz. The linear range of 5–100 µg/mL encompasses the clinically significant threshold for CRP-based cardiovascular risk stratification. This robust quantitative analysis confirmed the successful development of a highly effective electrochemical immunosensor for CRP detection.

Residual analysis was performed to validate the linear regression model by calculating the difference between measured and predicted Rct values at each CRP concentration (Figure 4e,f). At both 0.1 Hz and 1 Hz, the residuals were randomly distributed around zero (sum of residuals = −0.33 Ω and +0.51 Ω, respectively) with no systematic trend or curvature. These results indicate a good relationship between the experimental and fitted values, confirming the suitability of the semi-logarithmic linear calibration model for CRP quantification.

**Figure 5:**
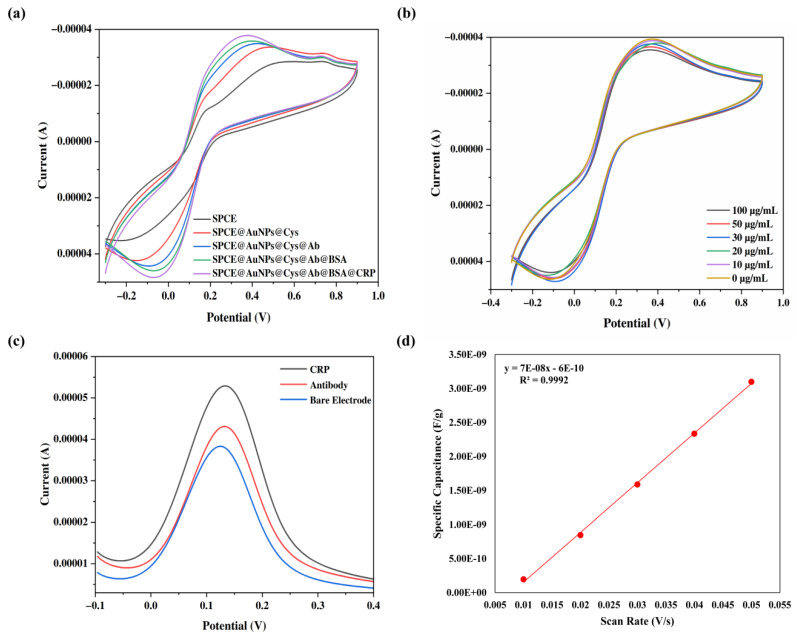
(**a**) CV curves of SPCE, SPCE@AuNPs@Cys, SPCE@AuNPs@Cys@Ab, SPCE@AuNPs@Cys@Ab@BSA, and SPCE@AuNPs@Cys@Ab@BSA@CRP at scan rates. (**b**) CV curves with different concentrations of CRP. (**c**) DPV curves of bare electrode (blue), antibody-modified electrode (red), and CRP-bound electrode (black). (**d**) Specific capacitance linear fitting curve of scan rate (V/s). Data are presented as mean ± standard deviation from three sets of data (*n* = 3).

The cyclic voltammograms of 5 mM [Fe(CN)_6_]^3−^/^4−^ in 0.1 M KCl were recorded in a potential range of −0.3 to 0.9 V. This potential window was selected to obtain well-defined and symmetric redox peaks for the [Fe(CN)_6_]^3−^/^4−^ redox couple. As shown in Figure 5a, systematic changes in the redox peak current were observed with each modification step. The bare screen-printed carbon electrode exhibited a baseline redox response. Following modification with highly conductive AuNPs and L-cysteine, a significant increase in peak current was observed, indicating enhanced electron transfer and an increase in the effective electroactive surface area. Subsequent immobilization of the non-conductive anti-CRP antibodies and blocking with BSA resulted in a gradual decrease in peak current, as these layers hindered electron transfer of the redox probe. The most pronounced decrease in current was observed after specific binding of the target CRP, confirming the formation of an insulating antigen–antibody complex on the electrode surface [21].

The analytical performance was evaluated using the concentration-dependent CV response (Figure 5b). The constructed immunosensor was tested against CRP concentrations (5–100 µg/mL). As CRP concentrations increased, both anodic and cathodic peak currents fell gradually. This decrease in current was produced by the formation of a thicker, more resistant layer of Ab@CRP immunocomplexes on the electrode surface, which gradually slowed the redox probe diffusion and electron-transfer kinetics. The constant and continuous current change across the concentration range confirmed the immunosensor capability for quantitative CRP detection, which was consistent with the trends seen in EIS data.

Figure 5c shows the DPV characterization of the electrode at successive fabrication stages (bare SPCE, anti-CRP Ab, and after CRP incubation), confirming that the conductive AuNP/chitosan scaffold maintains the electrochemical activity throughout the assembly process. The current values in Figure 5c reflected the cumulative electrochemical properties of the scaffold at each fabrication stage and are not intended to represent the quantitative dose–response relationship, which is established through EIS calibration (Figure 4) and DPV dose–response panel (Figure S4a). The substantial variations in DPV peak current at each stage of immunosensor construction proved the antibody immobilization and target analyte binding.

Figure 5d plots the specific capacitance (F/g) vs. the scan rate (V/s). The specific capacitance against scan rate curve describes the charge storage capacity of the modified electrodes, as determined by CV. The constructed immunosensor demonstrated electrochemical efficiency, as well as its stability, reproducibility, and ability to maintain performance across varying scan rates. The linear relationship observed shows that the specific capacitance increases with the scan rate. The high R^2^ value of 0.9992 confirmed the consistency and reliability of the proposed immunosensor (Figure 5d). The specific capacitance was computed using the following equation:

(2) $$\mathrm{Cs}\;=\;\frac{A}{2\;k\;m\;\Delta v}$$

where Cs denoted specific capacitance, A represented area under the curve, k denoted scan rate (V), m designated mass of the materials (g) and Δv denoted potential window.

Figure S3a shows cyclic voltammograms recorded at scan rates ranging from 0.01 to 0.05 V/s. The CV curves show a pair of well-defined redox peaks, corresponding to the oxidation and reduction reactions at the electrode surface, specifically confirming a surface-confined, adsorption-controlled electron-transfer process. A clear curve was observed, where both the anodic (oxidation) and cathodic (reduction) peak currents increased consistently with the scan rate [35]. The presence of distinct, well-shaped peaks further confirms the successful immobilization and electrochemical activity of the redox probe on the electrode surface. The effect of scan rate on the oxidation peak current was investigated in the range 0.01–0.05 V s^−1^. As shown in Figure S3b, the anodic peak current ($I_{pa}$) increased linearly with the scan rate (v), which was fitted by the regression equation $I_{pa}=0.0026\;v+2.0\times 10^{-5}$ (R^2^ = 0.9995). The nearly proportional increase of $I_{pa}$ with v, rather than with $v^{1/2}$, indicates that the electrochemical process is predominantly controlled by a surface-confined (adsorption-controlled) mechanism [36].

### 3.3. Analytical Performance of Immunosensor

The analytical performance of the developed electrochemical immunosensor was evaluated under optimized experimental conditions by monitoring the DPV response across a range of CRP antigen concentrations. As shown in Figure S4a,c, the decrease in DPV peak current with increasing CRP concentration is fully consistent with the steric blocking mechanism that also governs EIS Rct increase. Both observations reflected the same physical process: the antibody CRP immunocomplex forms an insulating protein layer that impedes the diffusion and electron transfer of the [Fe(CN)_6_]^3−^/^4−^ redox probe. The DPV current decrease and EIS Rct increase are the voltammetric and impedimetric read-outs, respectively, of the same blocking event [37].

In the sandwich HRP/TMB/H_2_O_2_ assay (Figure S4b,d), the signal is generated through enzymatic amplification. HRP-conjugated secondary antibodies are introduced to the electrode surface in proportion to the amount of captured CRP. As more CRP is captured, more HRP-labeled secondary antibody is assembled into the sandwich complex, resulting in greater HRP catalytic activity and a proportional increase in the DPV reduction current. HRP catalyzed the oxidation of TMB by H_2_O_2_, producing an electrochemically active species that is reduced at the electrode to generate a cathodic current. This mechanism is independent of the steric blocking model and operates through a distinct signal pathway from the label-free EIS and DPV modes [17,38].

The sensitivity of the developed immunosensor was assessed using LOD. We summarized and compared the analytical performance of the proposed immunosensor and previously reported sensors for CRP determination. The designed CRP immunosensors possessed significant advantages of low LOD as compared to previously reported immunosensors (Table 1). The calculated LOD was 0.16 µg/mL, which fell below the clinical reference range [39,40]. However, the detection limit achieved by the developed immunosensor is sufficient for detecting C-reactive protein in real samples. The proposed immunosensor exhibited a low LOD of 0.16 µg/mL, which is attributed to the synergistic effect of AuNPs-modified screen-printed carbon electrode platform. Unlike earlier sensor designs that depended on complex composites or random antibody adsorption, this platform employed a simple drop-casting of AuNPs onto the SPCE surface. As shown in Table 1, AuNP-modified SPCE achieved a limit of detection of 0.16 µg/mL, which was superior to most previously reported CRP sensors, including ssDNA/Au (3.12 µg/mL), Polytyramine/rGO (1.25 µg/mL), PCBGE/MWCNTs (0.74 µg/mL), SPGE (0.78 µg/mL), rGO/Ni/PtNPs (0.54 µg/mL), and G-SPE/PANI (0.5 µg/mL). Importantly, this sensitivity was achieved through a simpler fabrication strategy based on direct AuNP drop-casting, in contrast to complex nanocomposite systems such as rGO/Ni/PtNPs and MWCNTs that required multi-step synthesis. The enhanced performance of the immunosensor is increased by the high conductivity and catalytic activity of AuNPs, which promote faster electron transfer during electrochemical impedance spectroscopy measurements. This design also supported efficient antigen binding while minimizing non-specific interactions, thereby amplifying the impedance signal response. The combination of a disposable SPCE, AuNP-enhanced sensitivity, and a label-free electrochemical impedance spectroscopy readout represents the key innovation of this work and highlights its potential for practical point-of-care CRP detection. The LOD and limit of quantification (LOQ) were determined using the following formula:

(3) LOD = 3*σ*/*m*

(4) LOQ = 10*σ*/*m*

where *σ* is the standard deviation of the blank signal and slope *m* was taken from the semi-logarithmic calibration curve (Figure 4d). The blank response was measured using ten independently modified electrodes (*n* = 10), each assembled through the complete fabrication sequence and measured once in the absence of CRP (0 µg/mL) under standard EIS conditions. The use of independently modified electrodes ensures that *σ* captures inter-electrode fabrication variability, providing a conservative and realistic noise estimate. The LOQ was calculated to yield 0.86 µg/mL (0.1 Hz) and 0.55 µg/mL (1 Hz). LOQ values substantially confirm that the immunosensor provides reliable quantitative measurements across the entire claimed detection range.

### 3.4. Selectivity and Specificity

Accurate diagnostic procedures require the selective detection of specific analyte biomarkers. In this study, we selected common interferents, including ascorbic acid, citric acid, glucose, cysteine, glycine, urea, creatinine, matrix proteins (bilirubin, hemoglobin, and HSA), and inflammatory cytokines (IL-6, IgG, and TNF-α), based on their prevalence in biological samples and potential to affect the electrochemical signal. These compounds were chosen as they are commonly found in biological fluids such as serum, plasma, and urine, and could potentially affect the accuracy of point-of-care diagnostics. Interferents were tested at a 10-fold higher concentration than CRP to provide a controlled selectivity challenge and to evaluate whether common electroactive molecules, metabolic compounds, inflammatory cytokines, and serum proteins could produce non-specific impedance responses. Hence, it was crucial to evaluate how these interferences affect the sensor’s ability to specifically detect CRP without cross-reactivity. The significance of testing these interferents lies in ensuring sensor practical applicability in real-world clinical settings. The selectivity of the proposed immunosensor was evaluated by EIS using [Fe(CN)_6_]^3−^/^4−^ redox probe in the presence of CRP and interfering compounds. Figure S5 shows the change in charge transfer resistance at low frequency cutoffs of 0.1 Hz (Figure S5a) and 1 Hz (Figure S5b). Blank (water) exhibited a baseline ΔRct of approximately 430 Ω. In contrast, CRP induced a substantial increase in ΔRct, reaching approximately 1200 Ω, while the interfering substances showed considerably lower ΔRct values, typically below 900 Ω [35]. This indicated a strong blocking effect on the electron transfer of the ferri/ferrocyanide couple. The small error bars also indicate good repeatability of the measurements. Overall, these results confirm that the developed immunosensor produces the largest ΔRct for CRP, with interferents remaining below the CRP signal. Additionally, this immunosensor demonstrated good repeatability and robustness.

### 3.5. Stability and Reproducibility

**Figure 6:**
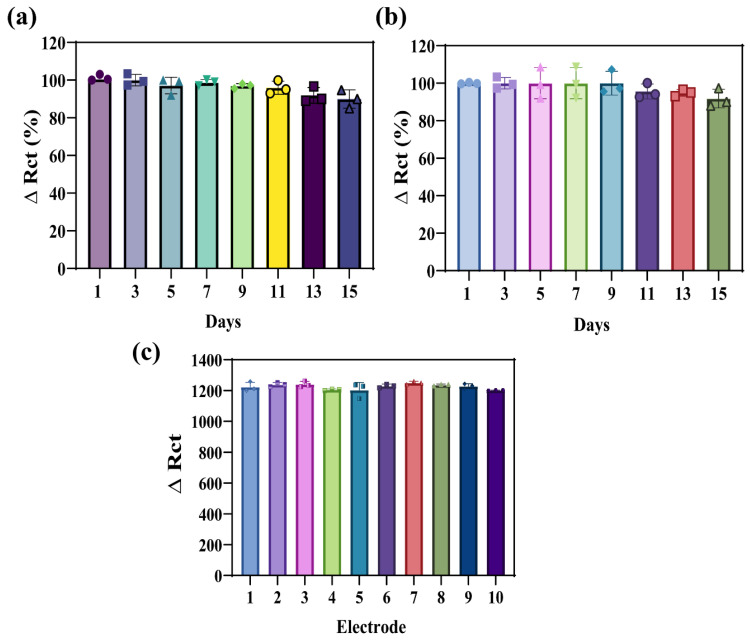
EIS results of CRP biosensor over 15 days. (**a**) Low frequencies 0.1 Hz and (**b**) 1 Hz, with 100 kHz as high frequency. (**c**) Reproducibility of immunosensor with ten identical electrodes. Data are presented as the mean ± standard deviation from three sets of data (*n* = 3).

The stability of the electrode used in the developed immunosensor was evaluated over a 15-day period by performing EIS with the [Fe(CN)_6_]^3−^/^4−^ redox probe. Figure 6a shows ΔRct (%) for CRP at a low frequency of 0.1 Hz, while Figure 6b shows the corresponding data at 1 Hz. Both figures clearly demonstrate that electrode response to CRP remained stable throughout the 15-day period, with minimal variation in ΔRct. The change in Rct for CRP remained over 95%, demonstrating the modified electrodes’ stability. The 15-day stability refers to short-term sensor performance under controlled storage, showing minimal degradation. This duration aligns with the early stages of sensor use, ensuring reliable performance during the initial weeks after manufacturing. These findings demonstrate that the constructed immunosensor is strong and stable, with no substantial decline in charge transfer resistance values up to 15 days. The small error bars emphasize the electrode’s remarkable repeatability across different days, increasing its reliability for long-term application in EIS-based detection. Corresponding reproducibility was further assessed by ten identical electrodes, which exhibited good reproducibility with a low relative standard deviation of less than 3.69% (Figure 6c). The presence of small error bars further underscores the high precision of the measurements across the different electrodes. The reproducibility observed in this study confirms the reliability and practical applicability of the developed CRP immunosensor for quantitative detection.

### 3.6. Effect of pH and Buffer on Immunosensor

The electrochemical performance of the immunosensor was examined within a pH range of 7.4 to 9.1 and different buffer systems, employing an [Fe(CN)_6_]^3−^/^4−^ redox couple as an electron-transfer mediator. This study aimed to evaluate the effect of pH and buffer environment on charge transfer resistance and antigen–antibody interaction dynamics. The corresponding current values of pH are visualized in the bar chart shown in Figure S6a. Measurements were conducted at a low-frequency cutoff of 0.1 Hz using the EIS method. The results indicated that a pH of 8.0 identified the highest ΔRct (927 Ω), signifying optimal conditions for CRP binding and signal transduction. Deviations from this pH, such as at pH 7.4 (675 Ω), pH 8.8 (555 Ω), and pH 9.1 (640 Ω), resulted in reduced ΔRct values, suggesting a decrease in the efficiency of CRP detection. Consequently, a pH of 8.0 was identified as the pH for the immunosensor’s optimal performance. At this pH, the immunosensor exhibited the highest sensitivity and stability, ensuring optimal antigen–antibody interactions and consistent signal detection. The optimization of buffer systems for the constructed immunosensor was also investigated by comparing across six different buffers—PBS, PIPES, HEPES, Tris, Borate, and MES—using the EIS method at a low-frequency cutoff of 0.1 Hz. The results in Figure S6b demonstrate that Tris buffer yielded the highest ΔRct (2020 Ω), indicating its superior ability to facilitate specific CRP binding and signal transduction, thereby offering optimal sensitivity. HEPES (1800 Ω) and MES (1700 Ω) also exhibited strong performances. Conversely, PBS (1400 Ω) and Borate (1430 Ω) buffers showed the lowest ΔRct values. Subsequently, Tris buffer was identified as the optimal buffer system for this immunosensor, offering superior stability and performance compared to other options. Its compatibility with immunosensor components and ability to maintain consistent pH and ionic strength make it the best choice for reliable immunosensor functionality. These results confirmed that the robust performance of the proposed immunosensor was elevated not only by nanomaterial design but also by systematic methodological optimization.

### 3.7. Regeneration Study of Immunosensor

**Figure 7:**
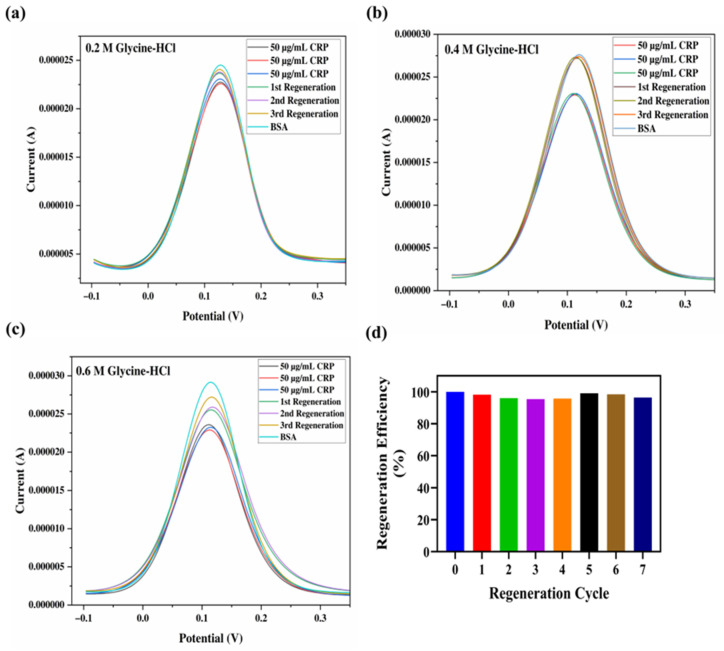
The effect of Glycine–HCl concentration on the reusability of CRP immunosensor. (**a**) 0.2 M, (**b**) 0.4 M, and (**c**) 0.6 M Glycine–HCl as the regeneration buffer. (**d**) Comparative bar chart showing the regeneration efficiency (%) of immunosensor over seven consecutive cycles using the optimized 0.4 M Glycine–HCl concentration. All measurements were performed with a fixed CRP concentration of 50 µg/mL.

The regeneration test evaluates the ability of an immunosensor to be reused by removing CRP from the immobilized antibody without damaging the antibody-binding sites. This feature is distinct among the electrochemical CRP sensors compared in Table 1. The regeneration performance was measured by the DPV technique in a 50 µg/mL CRP solution. Glycine–HCl was used as the regeneration agent with pH 3.0. The peaks for the first, second, and third regeneration cycles showed a slight but visible decrease in current compared to the initial CRP measurement when using 0.2 M Glycine–HCl (Figure 7a). This suggested that 0.2 M is not quite strong enough to remove all bound CRP. The peaks in Figure 7b are almost perfectly overlapping when using 0.4 M Glycine–HCl. The signal recovery is nearly 100% across all cycles, indicating that the sensor surface is completely cleaned and the antibodies remain fully functional. It provides enough chemical potential to dissociate the CRP–antibody complex completely while remaining mild enough that the antibody’s tertiary structure remains intact and functional. There is a significant and progressive drop in peak current with each cycle when using 0.6 M Glycine–HCl (Figure 7c). The peaks become inconsistent, indicating the excessive dissociation and damage of the antibody immobilization on the sensor surface. The bar chart in Figure 7d confirms the stability of the developed immunosensor, showing 97% efficiency even after seven cycles when using the optimized conditions, proving the robustness of the immunosensor.

### 3.8. Spiked Recovery Experiments in Human Serum Matrix

The applicability of the constructed immunosensor for CRP detection was investigated by dropping 10 µL of serum sample onto the immunosensor and incubating it for 50 min. The 50 min incubation time was optimized to ensure efficient antigen–antibody binding on the sensor surface. Preliminary experiments showed that shorter times lacked sufficient interaction, while longer durations did not improve binding but increased assay time. This 50 min period strikes a balance between optimal binding and practical real-time sensor use. Baseline CRP electrode was prepared through all fabrication steps up to BSA blocking. The electrode was rinsed gently with PBS. Then, 50 µL of redox probe solution containing 5 mM [Fe(CN)_6_]^3−^/^4−^ in 0.1 M KCl was dropped onto the electrode and the EIS spectrum was recorded under standard conditions with a frequency range of 0.1 Hz to 100 kHz and an amplitude of 0.005 V. CRP concentrations in the sample were established using the external standard method. As shown in Table 2, the recovery percentage and relative standard deviation values from three sensors (*n* = 3) ranged from 98.01% to 107.14% and 0.23% to 4.02%, respectively. The constantly high recovery percentage and low RSD values across all tested concentrations in the serum sample give a strong indication that the developed electrochemical immunosensor was not affected when human serum samples were used.

## 4. Conclusions

In this study, we successfully developed a fast, accurate, and label-free electrochemical immunosensor based on an AuNP-modified screen-printed carbon electrode for the sensitive and selective detection of CRP. The incorporation of AuNPs significantly enhanced the effective surface area and electrical conductivity of the electrode, providing a robust platform for electrochemical sensing. EIS revealed a strong linear correlation between charge transfer resistance and the logarithm of CRP concentration over a clinically relevant range of 5–100 µg/mL. This concentration range is critical for diagnosing mild to severe inflammatory conditions, including infections, chronic inflammatory diseases, and cardiovascular risk. The developed immunosensor demonstrated excellent specificity against common interfering species and showed reliable performance in human serum samples, confirming its applicability in real-world clinical settings.

In addition to high sensitivity and selectivity, the immunosensor exhibited good reproducibility, stability, and notable regeneration capability, enabling repeated use without significant loss of performance. A key contribution of this work lies in the systematic optimization of sensing conditions. While AuNP-modified immunosensors for CRP detection are well established, our results demonstrate that optimized conditions (Tris buffer, pH 8.0) significantly improved the electrochemical response compared to conventional PBS, leading to enhanced detection performance.

Overall, the proposed immunosensor combines simplicity, cost-effectiveness, and robust analytical performance, highlighting its strong potential for POC applications. Clinical validation using patient-derived samples spanning the full diagnostic range of CRP, including samples from individuals with confirmed inflammatory conditions, represents a critical priority for future work before the sensor can be considered for point-of-care deployment. Future work will focus on device miniaturization, performance enhancement, and scalability toward mass production, facilitating the broader adoption of this technology for field-based and at-home monitoring of inflammatory diseases.

## Figures and Tables

**Table 1:** Comparison of the analytical performance of other electrochemical immunosensors with the proposed immunosensor.

Material	Measurement Principle	Linear Range (µg/mL)	LOD (µg/mL)	LOD Method	Type of Sample	Regeneration Study	Reference
ssDNA/Au electrode	EIS	3.125–25	3.12	-	Serum	No	[26]
PCBGE/MWCNTs	DPV	1.25–80	0.74	3 s/m	Blood	No	[7]
Polytyramine/rGO	EIS/DPV	1.09–100	1.25	-	Serum	No	[41]
Magnetic rGO/Ni/PtNPs micromotors	Amperometry	2–100	0.80	S/N = 3	Serum/ plasma	No	[40]
rGO/Ni/PtNPs	Amperometry	1–100	0.54	S/N = 3	Serum/ plasma	No	[42]
SPGE	EIS	6.25–50	0.78	3σ/m	Serum	No	[43]
Graphite electrode/poly-3 aminothiophenol	CV/DPV	0.075–150	7.24	-	Serum	No	[44]
AuNP	Colorimetric	0.9–20.7	1.2	tS_y_/r	Serum	No	[45]
G-SPE/PANI	EIS	0.25–2	0.5	3σ/m	FBS	No	[46]
Aptamer probes	FET	0.1–50	0.14	3.3σ/m	PBS	No	[47]
Fab fragment	Fluorescent	5–20	1.58	-	Serum	No	[48]
PAAI	Colorimetric	3.15–100	1.15	-	Serum	No	[49]
AuNPs/SPCE	EIS	5–100	0.16	3σ/m	Serum	Yes (97%)	This work

**Table 2:** Spiked recovery data of electrochemical immunosensor for CRP detection.

Sample No.	Spiked CRP Concentration (µg/mL)	Measured Concentration (µg/mL)	(%) Recovery	(%) RSD
1	5	5.32	106.40	3.39
2	10	10.63	106.30	1.83
3	20	19.60	98.00	3.19
4	30	30.00	100.00	4.02
5	50	50.15	100.30	2.23
6	100	101.74	101.74	0.23

## Data Availability

Data is contained within the article.

## Supplementary Materials

The following supporting information can be downloaded at https://www.mdpi.com/article/10.3390/bioengineering13060592/s1, Figure S1: TEM and FESEM characterization of AuNPs. (a) TEM images at 100, 50, and 20 nm. (b) FESEM images of AuNPs 500, 200, and 100 nm magnifications; Figure S2: Characterization of bare citrate-capped AuNPs. (a) XPS spectrum. (b) FTIR spectrum. (c) Zeta potential measurement (d) DLS (e) EDS and (f) UV-vis absorption spectra of bare AuNPs (black curve) and AuNPs conjugated with CRP (red curve); Figure S3: Cyclic voltammograms recorded at different scan rate (V/s). (a) Scan rate study within range of 0.01–0.05 V/s. (b) Calibration curve of oxidation peak current at different scan rates V/s. Data are presented as mean ± standard deviation from three sets of data (*n* = 3); Figure S4: (a) DPV curves acquired at different concentrations of CRP in PBS containing 5 mM [Fe (CN)_6_]^3−^/^4−^. (b) DPV curves obtained at CRP concentrations (0, 10, 20, 30, 50, and 100 μg/mL) in electrolyte solution containing (0.003%) H_2_O_2_/ and (0.5 mg) 3,3′,5,5′-Tetramethylbenzidine (TMB). (c) The calibration plot of current against CRP concentration. (d) Corresponding linear calibration curve between the peak current and the CRP concentrations (0, 10, 20, 30, 50, and 100 μg/mL). Data are presented as mean ± standard deviation from three sets of data (*n* = 3); Figure S5: Electrochemical response of interfering compounds. (a) Selectivity of different interfering compounds at low frequencies cutoff 0.1 and (b) 1 Hz with 100 kHz as a high frequency. Data are presented as the mean ± standard deviation from three sets of data (*n* = 3); Figure S6: (a) Optimization of pH for CRP detection via EIS. (b) Effect of various buffer systems on change in charge transfer resistance. Data are presented as the mean ± standard deviation from three sets of data (*n* = 3); Table S1: Extrapolated parameters of the modified Randles equivalent circuit when fit to the measured Nyquist plots of bare and other modified electrodes as shown in Figure 3.
