# Supplementary material for: Chitosan-Modified Gold Nanoparticle-Based Electrochemical Immunosensor for C-Reactive Protein Detection
Source: Bioengineering (Basel). 2026 May 22;13(6):592. doi: 10.3390/bioengineering13060592 (PMC13295841; doi:10.3390/bioengineering13060592)
Supplement: Supplementary file 1 [file bioengineering-13-00592-s001.zip › bioengineering-4316219-supplementary.pdf]

Supplementary Materials

Chitosan-Modified Gold Nanoparticle-Based Electrochemical Immunosensor for C-Reactive Protein Detection

Bilal Ahmad 1,2, Changyun Quan 2, Xiyue Zhang 2, Haiyan Xia 2, Zhenhong Yuan 2, Chenghua Zhu 2, Yang Zhang 2, Haixia Yang 2,3, Xueqin Huang 2, Chunyi Tong 1, Bin Liu 1,* and Binjie Xu 2,*

Author Affiliations

1College of Biology, Hunan University, Changsha 410000, China

2Cofoe Medical Technology Co., Ltd., No. 87, Section 1, Huanbao East Road, Changsha 410000, China

3Hunan Provincial Key Laboratory of Micro & Nano Materials Interface Science, College of Chemistry and Chemical Engineering, Central South University, Changsha 410000, China

*Corresponding authors: binliu2001@hotmail.com (B.L.); binjie.xu@outlook.com (B.X.)

*Correspondence:

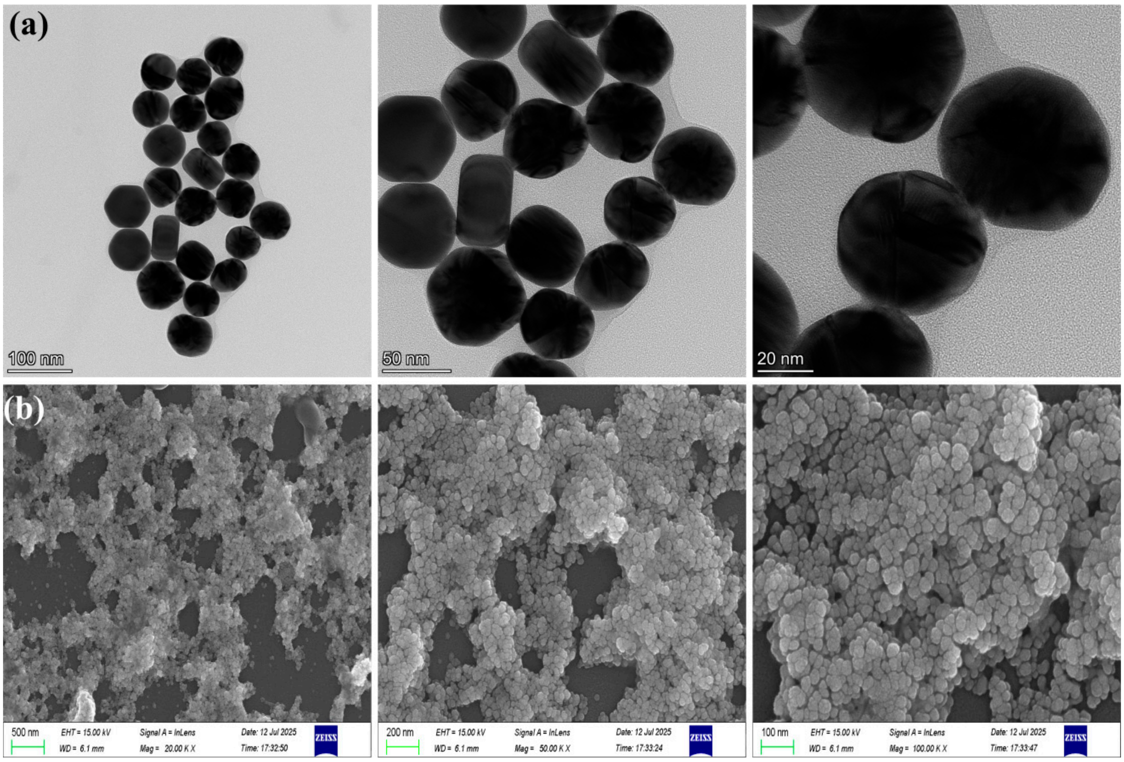

Figure S1. TEM and FESEM characterization of AuNPs. (a) TEM images at 100, 50, and 20 nm. (b) FESEM images of AuNPs 500, 200, and 100 nm magnifications.

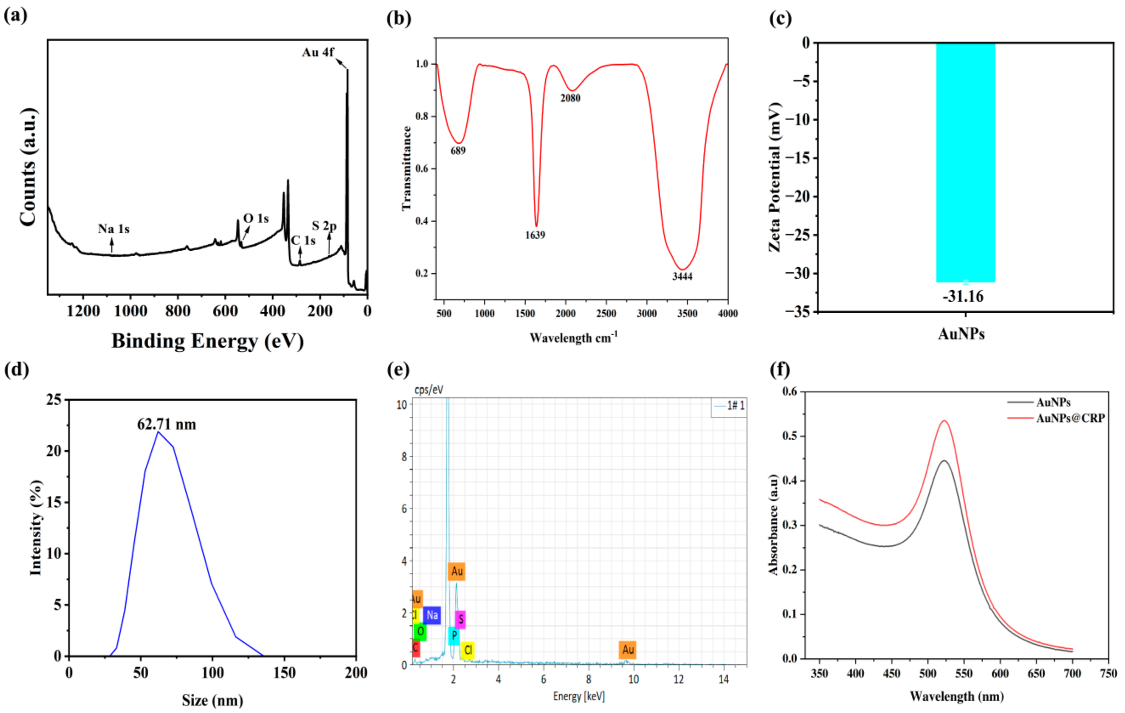

Figure S2. Characterization of bare citrate-capped AuNPs. (a) XPS spectrum. (b) FTIR spectrum. (c) Zeta potential measurement (d) DLS (e) EDS and (f) UV-vis absorption spectra of bare AuNPs (black curve) and AuNPs conjugated with CRP (red curve).

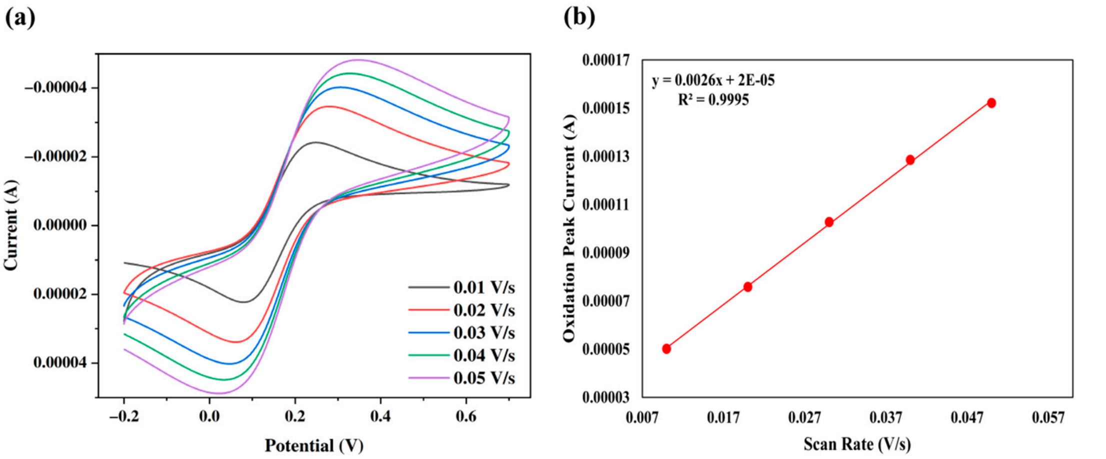

Figure S3. Cyclic voltammograms recorded at different scan rate (V/s). (a) Scan rate study within range of 0.01-0.05 V/s. (b) Calibration curve of oxidation peak current at different scan rates V/s. Data are presented as mean ± standard deviation from three sets of data (n = 3).

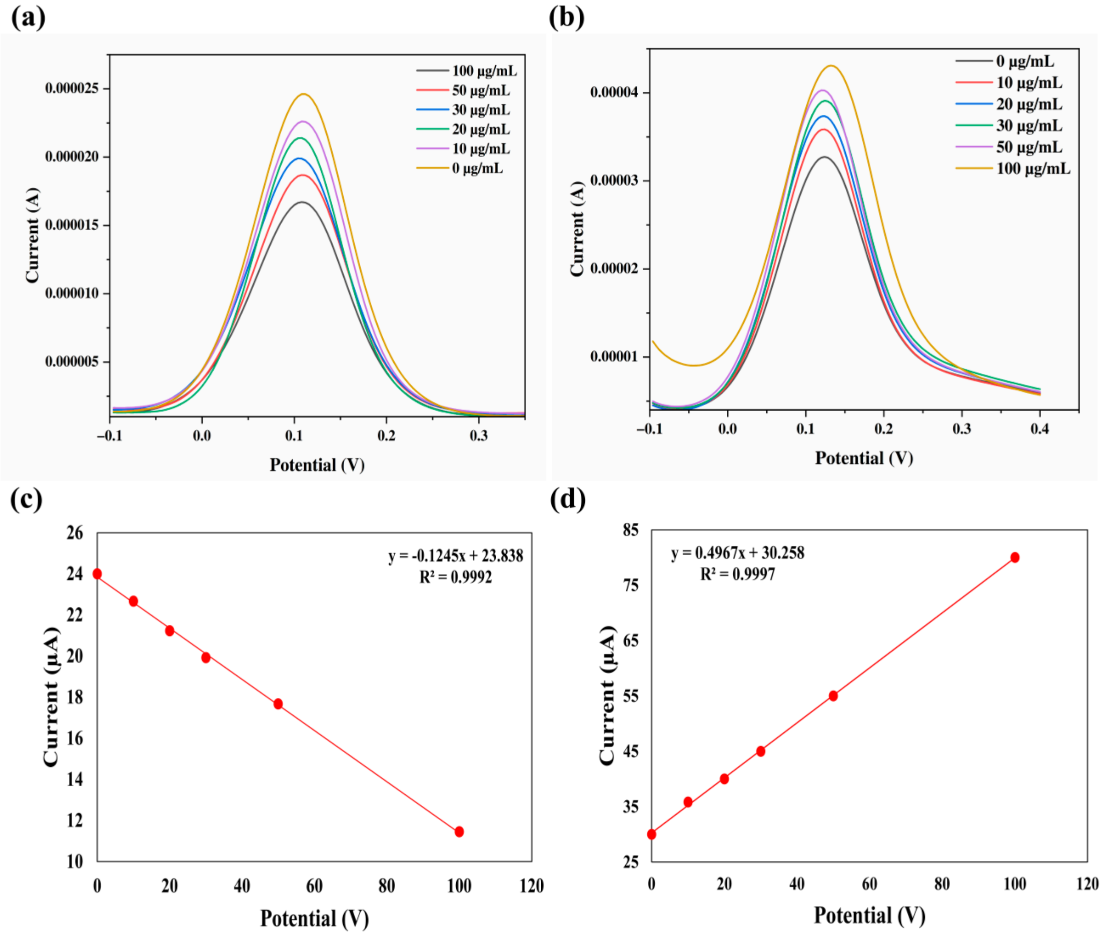

Figure S4. (a) DPV curves acquired at different concentrations of CRP in PBS containing 5 mM $[Fe(CN)_6]^{3-/4-}$. (b) DPV curves obtained at CRP concentrations (0, 10, 20, 30, 50, and 100 μg/mL) in electrolyte solution containing (0.003%) H_2O_2/ and (0.5 mg) 3,3',5,5'-Tetramethylbenzidine (TMB). (c) The calibration plot of current against CRP concentration. (d) Corresponding linear calibration curve between the peak current and the CRP concentrations (0, 10, 20, 30, 50, and 100 μg/mL). Data are presented as mean ± standard deviation from three sets of data (n = 3).

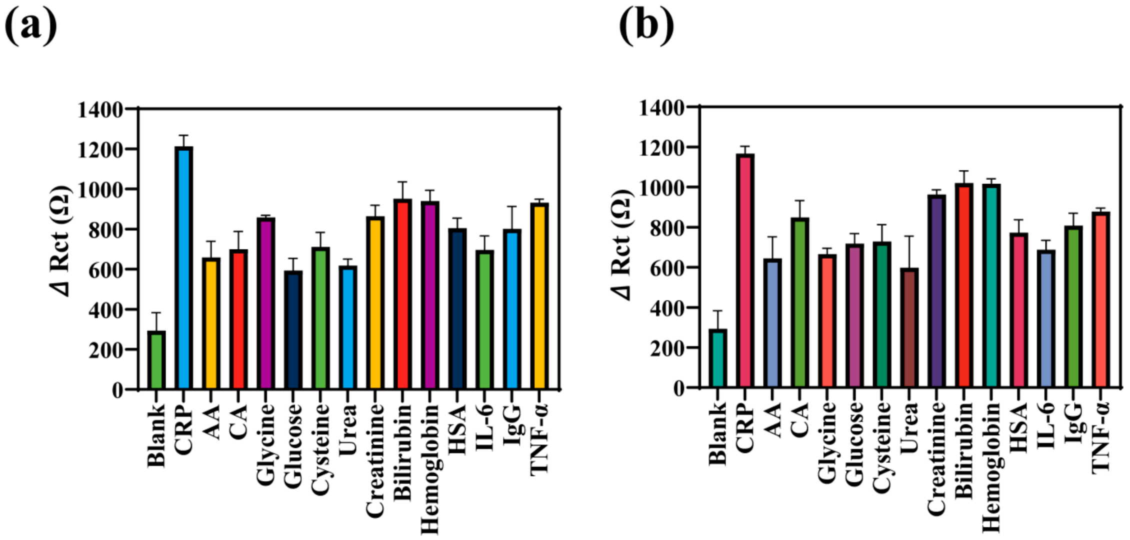

Figure S5. Electrochemical response of interfering compounds. (a) Selectivity of different interfering compounds at low frequencies cutoff 0.1 and (b) 1 Hz with 100 kHz as a high frequency. Data are presented as the mean ± standard deviation from three sets of data (n = 3).

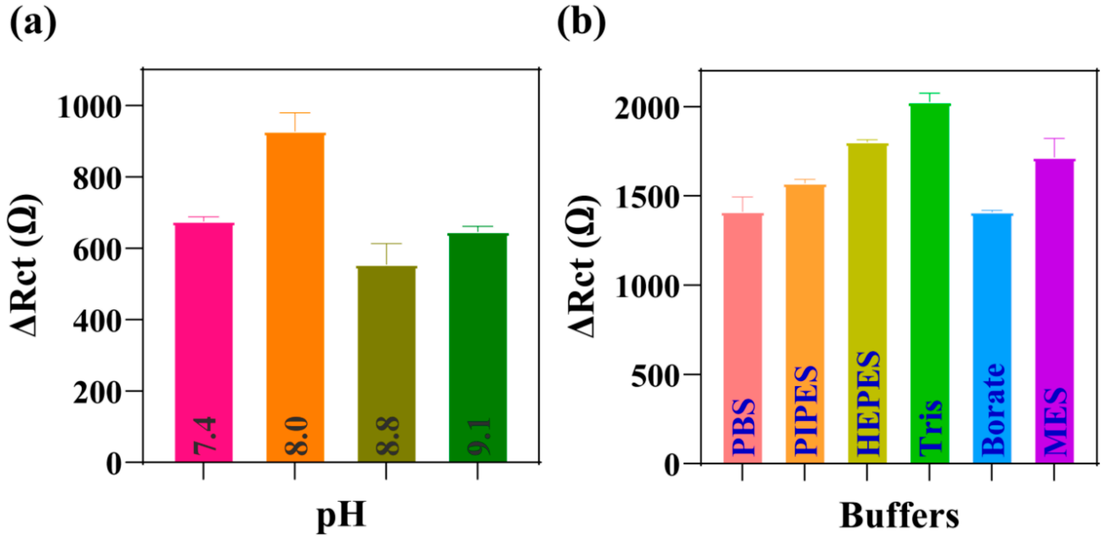

Figure S6. (a) Optimization of pH for CRP detection via EIS. (b) Effect of various buffer systems on change in charge transfer resistance. Data are presented as the mean ± standard deviation from three sets of data (n = 3).

Table S1: Extrapolated parameters of the modified Randles equivalent circuit when fit to the measured Nyquist plots of bare and other modified electrodes as shown in Figure 3.

**Electrode**	**Rct (Ω)**	**Rs (Ω)**	**CPE-T (Ω^{-1} s^n)**	**CPE-n**	**χ^2**
AuNPs	494	374.2	1.8405×10^{-5}	0.8512	0.0004277
AuNPs/Cys	587	365.4	1.3327×10^{-5}	0.8258	0.0008513
AuNPs/Cys/Ab	653	396.2	1.8334×10^{-5}	0.8552	0.0005194
AuNPs/Cys/Ab/BSA	716	408.7	1.8507×10^{-5}	0.8465	0.0003491
AuNPs/Cys/Ab/BSA/CRP	803	432.4	3.0285×10^{-5}	0.7681	0.0003179
